# Safety and Effectiveness of Neuro-thrombectomy on Single compared to Biplane Angiography Systems

**DOI:** 10.1038/s41598-020-60851-4

**Published:** 2020-03-11

**Authors:** Adrien Guenego, Pascal J. Mosimann, Max Wintermark, Jeremy J. Heit, Kevin Zuber, Tomas Dobrocky, Jean Albert Lotterie, Patrick Nicholson, David G. Marcellus, Jean Marc Olivot, Nestor Gonzalez, Raphaël Blanc, Vitor Mendes Pereira, Jan Gralla, Johannes Kaesmacher, Robert Fahed, Michel Piotin, Christophe Cognard, The RADON Investigators, Eike Piechowiak, Pasquale Mordasini, Felix Zibold, Celina Ducroux, Fabrice Bonneville, Jean Darcourt, Ivan Vukasinovic, Anne Christine Januel, Sylvie Monfraix, Caterina Michelozzi, Philippe Tall, Mikael Mazighi, Jean-Philippe Desilles, Gabriele Ciccio, Stanislas Smajda, Hocine Redjem, Benjamin Maier, Blake W. Martin, Elisa Guenego, Fanny Carbillet

**Affiliations:** 1 Interventional and Diagnostic Neuroradiology Department, Toulouse University Hospital, Toulouse, France; 2 Interventional and diagnostic Neuroradiology, Bern, Switzerland; 3 Interventional and Diagnostic Neuroradiology, Stanford Medical Center, California, USA; 4 Statistics department, Fondation Ophtalmologique Adolphe de Rothschild, Paris, France; 5 Stereotaxic Neurosurgery Department, Toulouse University Hospital, Toulouse, France; 6 Interventional and Diagnostic Neuroradiology, Toronto Hospital, Toronto, Canada; 7 Vascular Neurology, Stroke Department, Toulouse University Hospital, Toulouse, France; 8 Neurosurgery and Interventional Neuroradiology Department, Cedars Sinai Hospital, Los Angeles, USA; 9 Interventional Neuroradiology, Fondation Ophtalmologique Adolphe de Rothschild, Paris, France; 10 Interventional Neuroradiology Department, Clinical Center of Serbia, Serbia, Serbia; 11 Radiophysic Department, Toulouse University Hospital, Toulouse, France; 12 Anesthesiology Department, Toulouse University Hospital, Toulouse, France; 13 ALARA Expertise, Radiophysic Department, Strasbourg, France

**Keywords:** Health policy, Public health, Neurological disorders

## Abstract

An increasing number of centers not necessarily equipped with biplane (BP) angiosuites are performing mechanical thrombectomy (MT) in acute ischemic stroke patients. We assessed whether MT performed on single-plane (SP) is equivalent in terms of safety, effectiveness, radiation and contrast agent exposure. Consecutive patients treated by MT in four high volume centers between January 2014 and May 2017 were included. Demographic and MT characteristics were assessed and compared between SP and BP. Of 906 patients treated by MT, 576 (64%) were handled on a BP system. After multivariate analysis, contrast load and fluoroscopy duration were significantly lower in the BP group [100vs200mL, relative effect 0.85 (CI: 0.79–0.92), p = 0.0002; 22 vs 27 min, relative effect 0.84 (CI: 0.76–0.93), p = 0.0008, respectively]. There was no difference in recanalization (modified Thrombolysis-In-Cerebral-Infarction 2b-3), good clinical outcome (modified Rankin Scale 0–2), complications rates, procedure duration or radiation exposure. A three-vessel diagnostic angiogram performed prior to MT led to a significant increase in procedure duration (15% increase, p = 0.05), radiation exposure (33% increase, p < 0.0001) and contrast load (125% increase, p < 0.0001). Mechanical neuro-thrombectomy seems equally safe and effective on a single or biplane angiography system despite increased contrast load and fluoroscopy duration on the former.

## Introduction

Multiple randomized controlled trials have demonstrated the benefits of cerebrovascular mechanical thrombectomy (MT) in acute ischemic stroke patients with emergent large vessel occlusion (AIS-ELVO)^1–6^. Two further randomized trials evaluating late-onset strokes with favorable perfusion imaging^7,8^, have broadened its indications^9,10^. Adequate training is necessary for these procedures, which are often more complicated than may be anticipated^11,12^. Most neuro-interventionalists prefer working on biplane (BP) angiosuites since two simultaneous projections of the material and anatomical structures per contrast injection tends to reduce the risk of arterial perforation compared to single plane (SP) procedures^12,13^. Nonetheless, X-ray and contrast agent exposure remain a major public health concern in terms of carcinogenesis^14–17^, risk of renal failure^18–20^ or toxic reactions, respectively^21,22^.

Since an increasing number of centers are performing MT^12^ in the context of ELVO without necessarily being equipped with BP angiosuites, there is a need to understand whether MT performed on SP is equivalent in terms of safety, effectiveness, radiation and contrast agent exposure^22^.

Since evidence regarding safety and effectiveness of MT on SP and BP is contradictory^18,23–25^, we performed a multicentric analysis based on prospectively acquired data from four internationally accredited comprehensive stroke centers to establish which variables are associated with an increased radiation or contrast exposure during MT.

## Results

### Baseline characteristics

Of the 906 MT included, 330 (36%) and 576 (64%) were performed on SP and BP systems, respectively. Baseline characteristics did not differ significantly (see Table 1 for details) for clinical, imaging and interventional characteristics. For additional details, please check on-line supplemental material. Mean age was 69 ± 15 years (range: 18–100). Median National Institute of Health Stroke Scale (NIHSS) at admission was 17 (Interquartile range or IQR 12–21).

**Table 1:** Description of single and biplane groups.

Angioroom Type	All n (%)	Single-plane Imaging n (%)	Biplane Imaging n (%)
Number (Total 906)	906 (100)	330 (36.4)	576 (63.6)
Center*			
A	126	38 (11.5)	88 (15.3)
B	488	179 (54.2)	309 (53.6)
C	151	0 (0)	151 (26.2)
D	141	113 (34.2)	28 (4.9)
Dates*			
January-December 2014	207 (23)	119 (36)	88 (15)
January-December 2015	462 (51)	157 (48)	305 (53)
January 2016-May 2017	237 (26)	54 (16)	183 (32)
Gender			
Male	444	157 (52.4)	287 (49.8)
Female	462	173 (47.6)	289 (50.2)
Age (years): Median, Q1, Q3*	72 (59–81)	69 (58–80)	72 (59–82)
Weight (kg): Median, Q1, Q3	75 (65–85)	75 (63–84)	75 (65–86)
Pre-stroke mRS: Median, Q1, Q3	0 (0–1)	0 (0–0)	0 (0–1)
Onset NIHSS: Median, Q1, Q3	17 (12–21)	17 (11–21)	17 (12–20)
Supra-Aortic imaging			
Yes	572	208 (63)	364 (63.2)
No	334	122 (37)	212 (36.8)
Side			
Right	375	131 (40)	244 (42.4)
Left	465	174 (53)	291 (50.5)
Midline (Basilar artery)	66	25 (7)	41 (7.2)
Level of Occlusion			
MCA-M1	473	170 (51.5)	303 (52.6)
MCA- M2	117	38 (11.5)	79 (13.7)
Terminal ICA	157	60 (18.2)	97 (16.8)
Tandem	93	37 (11.2)	56 (9.7)
Basilar artery	66	25 (7.6)	41 (7.1)
Intravenous thrombolysis			
Yes	639	257 (77.9)	382 (66.3)
No	267	73 (22.1)	194 (33.7)
Anesthesia*			
General (GA)	307	131 (39.7)	176 (30.6)
Conscious Sedation (CS)	578	194 (58.8)	384 (66.7)
CS converted in AG	21	5 (1.5)	16 (2.8)
Pre-intervention angiogram*			
Yes	609	279 (84.5)	330 (57.3)
No	297	51 (15.4)	246 (42.7)
Technique*			
Stent-retriever	374	188 (57)	186 (32.3)
Aspiration	348	120 (36.4)	228 (39.6)
Combined	184	22 (6.7)	162 (28.1)
Stenting and/or angioplasty			
Yes	129	52 (15.8)	77 (13.4)
No	777	278 (84.2)	499 (86.6)
Stroke etiology*			
Cardio-embolic	423	106 (48.9)	317 (57.9)
Atherosclerosis	126	39 (18)	87 (15.9)
Dissection	38	13 (6)	25 (4.6)
Other	178	59 (27.2)	119 (21.7)
Stroke onset to groin (min): Median (Q1, Q3)	244 (230–275)	254 (218–282)	239 (226–273)

^*^Significant difference between groups, p < 0.05, used in the multivariate regression.

BP: Biplane angiosuite.

CS: Conscious Sedation.

GA: General Anesthesia.

MCA-M1: First portion of the Middle Cerebral Artery.

MCA-M2: Second portion of the Middle Cerebral Artery.

MT: Mechanical Thrombectomy.

N: Number.

NIHSS: National Institute of Health Stroke Scale.

Q1: First quartile.

Q3: Third quartile.

SP: Single-Plane/Mono-plane angiosuite.

TICI: Thrombolysis In Cerebral Infarction score.

### Procedure duration

All procedures were performed within 6 hours of stroke onset according to international recommendations^26^.

Time from stroke onset to groin puncture as well as procedure time (from initial groin puncture to last image in minutes) are described for all patients and did not differ significantly.

### Statistical analysis

After multivariate analyses, there was no significant difference in rates of successful recanalization, periprocedural complication, nor for DAP or air kerma (Tables 1 and 2), whereas contrast load and fluoroscopy duration remained significantly lower on BP compared to SP interventions [100 vs 200 mL (50% decrease, relative effect 0.85,CI: 0.79–0.92, p = 0.0002) and 22 vs 27 min (19% decrease, relative effect 0.84, CI: 0.76–0.93, p = 0.0008), respectively].

**Table 2:** Uni and multivariate results of the single versus biplane comparison.

Angioroom Type	Single-plane Imaging n (%)	Biplane Imaging n (%)	p -value univariate	p-value multivariate
Number (906 total)	330 (36.4)	576 (63.6)		
Recanalization rate (TICI 2b-3)	280 (84.8)	480 (83.3)	0.57	0.53
Complication rate %	52 (15.8)	89 (15.4)	0.92	0.54
Procedure delay (min): Median, Q1, Q3	45 (30–71)	44 (30–73)	0.64	0.72
24h NIHSS: Median, Q1, Q3	11 (4–19)	12 (4–20)	0.47	0.47
3 months good mRS (0–1–2)	165 (50.0)	231 (40.1)	<0.0001	0.22
DAP Gy.cm²: Median, Q1, Q3	170 (106–280)	140 (92–235)	0.0001	0.57
Kerma Gy: Median, Q1, Q3	0.96 (0.6–1.9)	0.97 (0.6–1.7)	0.38	0.13
Contrast load mL: Median, Q1, Q3	200 (150–250)	100 (60–180)	<0.0001	0.0002
Scopy duration min: Median, Q1, Q3	27 (17–41)	22 (14–37)	0.00012	0.0008

BP: Biplane angiosuite.

cm.²: centimeter square.

DAP: Dose–Area Product.

Gy.cm²: Gray per centimeter square.

mL: milliliter’s.

mRS: modified Rankin Scale.

MT: Mechanical Thrombectomy.

N: Number.

NIHSS: National Institute of Health Stroke Scale.

Q1: First quartile.

Q3: Third quartile.

SP: Single-Plane/Mono-plane angiosuite.

TICI: Thrombolysis In Cerebral Infarction score.

There was no significant difference in rates of good clinical outcome (mRS 0–2) between BP and SP (40.1% versus 50%, p = 0.22 after multivariate analyses).

### Factors associated with increased dose or contrast

Three variables were independently associated with an increased DAP, kerma and contrast load, namely cervical dissection as ELVO etiology (207 Gy.cm² vs 148 for other stroke etiology p < 0.0001), tandem occlusion (239 Gy.cm² vs 148 for other occlusion types p < 0.0001) or the need for stenting and/or angioplasty (271 Gy.cm² vs 137 without angioplasty or stenting, p < 0.0001). Use of a stent-retriever and general anesthesia were associated with increased radiation dose in univariate but not in multivariate analyses.

After adjustment for potential confounders, a three or four vessel diagnostic cerebral angiogram before MT significantly increased radiation dose: DAP 161 Gy.cm² vs 122 without diagnostic angiograms (33% increase, p < 0.0001). The same effect was observed for contrast load and procedure duration: 180 vs 80 mL (125% increase, p < 0.0001) and 46 vs 40 minutes (15% increase, p = 0.05), respectively, without affecting the rates of successful recanalization, periprocedural complication or good clinical outcome.

## Discussion

According to our multicentric data, MT performed on BP does not improve efficacy or safety compared to SP interventions, despite the added subjective sense of comfort acknowledged by the majority of operators (which was not specifically measured).

Overall, biplane use significantly reduced contrast load (50% decrease, p < 0.0001) and fluoroscopy duration (19% decrease, p = 0.0056). DAP and kerma, however, were not affected after adjusting for potential confounders, contrary to the effect of radiation reduction dose-systems as showed in the literature^27^.

Likewise, rates of successful recanalization, periprocedural complications, good functional outcome and total procedure duration did not differ between BP and SP interventions. As expected, more complex and time-consuming interventions, such as those associated with cervical dissection, tandem occlusions^28^, stenting and/or angioplasty were associated with increased contrast load and radiation doses. Use of stent-retriever, contact aspiration^29^, general anesthesia or conscious sedation^30^ were associated with similar rates of safety and effectiveness. Despite increased radiation exposure with stent-retrievers use or general anesthesia in univariate analyses, this was no longer significant after multivariate analysis.

Our results contradict a recent literature review suggesting reduced radiation, contrast load, risk of complications and procedure duration with biplane angiosuites^13^. As recognized by the authors of the latter, however, the level of evidence was low. Moreover, MT was not the sole intervention studied, as opposed to the data present. It is noteworthy that neurointerventional procedures^31^, when compared to coronary angioplasty^18^ and angiography^32^, may be associated with a lower DAP on BP systems.

Based on the higher acquisition and maintenance costs of BP systems and the absence of an obvious clinical benefit for MT^13^, there is currently no evidence to recommend performing such procedures exclusively on BP or to withhold MT from SP systems. One could even argue that the latter appears to be more cost-effective and that the number of treatment sites capable of offering MT could be expanded if BP is not mandatory. One should emphasize, however, that our data was acquired only in high-volume academic centers with expertly trained operators, meaning our results do not necessarily apply to other clinical settings^33^.

Although some physicians believe that a three or four vessel diagnostic cerebral angiogram, including both internal carotid arteries and at least the dominant vertebral artery, is essential before performing MT, mainly to assess all collateral flow routes^34^, our results indicate that this may be more harmful by increasing contrast load, procedure duration and radiation exposure unnecessarily. Although the additional diagnostic runs do not negatively influence clinical outcome, collateral flow is nowadays sufficiently well assessed on admission computed-tomography or magnetic-resonance-imaging, especially when advanced imaging such as multiphase computed-tomography angiogram or perfusion studies are performed^35^. Moreover, additional diagnostic runs are unlikely to modify the therapeutic approach in the vast majority of situations, since thrombectomy will be attempted as long as an ELVO is still present. Lastly, the cumulative radiation dose from DSA, computed-tomography, computed-tomography perfusion and angiography prior to MT should be minimized as much as possible.

Our study is the first to compare SP and BP with such a high number of patients, international centers and focusing on a large variety of both technical and clinical outcomes. Our approach let us describe a game-changing result in the assessment of collateral flow, showing that invasive assessment of a four vessel diagnostic cerebral angiogram before a MT procedure could be more harmful for patients than useful.

We do believe the SP versus BP comparison is a major issue when more and more centers tend to treat patients for MT. Whether facilities without biplane systems are able to perform MT whether the installation of a biplane system is required are major organizational issues.

Lastly, on the contrary to a recent similar analysis^36^, our results emphasized the importance of both AP and lateral views in interventional neuroradiology, showing a complete different technical approach than Friedrich&al. Their results showed a similar contrast load with twice the radiation dose for biplane angiosuites, implying they performed the same number of runs on SP and BP: the second view was not useful for them. In these circumstances, rates of complication and successful recanalisations they describe may be difficult to grasp and interpret knowing the low level of evidence of a study with less than 200 patients.

### Limitations

There were several limitations due to the retrospective nature of our study. Exposure related to flat panel computed tomography following MT completion (mainly to evaluate vessel perforation in selected cases) may have unduly increased the mean DAP. Given the low total number of complications, however, this appears to be insignificant. Secondly, the cumulative dose of patients screened with CT at admission was not incorporated in our analyses, although the proportion screened with this modality in the BP and SP population were similar. Thirdly, while individual skills, experience, speed and radioprotection knowledge may differ from one center to the next or within a given institution and influence the results, it is unlikely to have played a significant role considering the similar experience, fellow involvement and interventional protocols in the four participating comprehensive stroke centers. Fourthly, estimation of reperfusion success, self-assessed by non-core-lab adjudicated mTICI evaluations, may have incorporated suboptimal single-plane evaluations with possible missing antero-posterior or lateral views before and after recanalization. Fifthly, the SP and BP arms were unbalanced and not primarily randomized to study their performance for MT, our results may then be limited, randomized trials would be necessary to assess this matter. A relatively high number of patients between 2015 and 2017 were treated on upgraded angiosuites equipped with dose reduction technologies, they were not included in the present study to avoid bias in radiation doses analyses, the impact of dose reduction technology was not evaluated. The scale to report the amount of contrast agent administered was not specifically described for each center however, inclusion of centers in the multivariate analysis may have limited bias. Lastly, number of attempts and number of devices used for MT were not reported in our study.

## Conclusion

Mechanical neuro-thrombectomy seems equally safe and effective on a single or biplane angiography system, despite increased contrast load and fluoroscopy duration on the former, especially if a three or four vessel pre-interventional diagnostic angiogram is performed.

## Materials and Methods

The study protocol was approved by the institutional review board of each of the four certified comprehensive stroke centers in France, Switzerland and the United States of America (See Supplemental material). Patient informed consent was waived for this retrospective analysis of pooled anonymized prospectively acquired data. Adherence to the STROBE criteria^37^ and compliance with the Health Insurance Portability and Accountability Act were enforced.

### Population

All consecutive patients who underwent MT for AIS-ELVO confirmed by magnetic-resonance-imaging or computed-tomography^38,39^ between January 2014 and May 2017 on a biplane (BP) or single-plane (SP) angiosuite were included.

Functional outcome using the modified Rankin Scale (mRS)^40^ was assessed at 90 days by a certified stroke neurologist.

Over the 3-year study period, fellow involvement ranged from assistance to supervised or fully independent MT but was not recorded.

### Dose metrics

Variables assessed were Kerma (Gy), dose-area product (DAP) (Gy.cm²) and total fluoroscopy time (minutes).

### Imaging system parameters

The technical parameters of each angiosuite (see Supplement Tables 1 and 2) were reported and verified by two radio-physicists (F.C. and S.M.). A 27 or 32 cm field-of-view was used for the vast majority of treatments. DAP, kerma and fluoroscopy duration were extracted from the dose reports of the picture archiving systems by one of the local interventional neuro-radiologist or medical physicist. Angiosuites were regularly calibrated and controlled according to national and international standards.

### Statistics/data analysis

The primary outcome was the rate of good functional outcome at 90 days (defined as a mRS of 0 to 2) dichotomized into SP and BP interventions.

Secondary outcome measures were rates of successful recanalization (defined as modified Thrombolysis In Cerebral Infarction score [mTICI] 2b or 3), complications (periprocedural perforation, hemorrhage, and iatrogenic thromboembolic events), procedure and fluoroscopy time (from initial groin puncture to last image), total radiation dose (air kerma and DAP) and contrast agent volume injected in SP versus BP then depending on the angiosuite brand. Furthermore, we assessed which variables were associated with an increased radiation or contrast load during MT.

Since MT focuses on the head and neck region, we assumed there was no need to correct for automated weight-adapted variation of radiation dose, as occasionally described^41–43^. Nonetheless, the absence of interaction with all variables was statistically tested.

A descriptive analysis was first performed. Categorical variables were summarized using frequency, individual and cumulative percentages. Continuous variables were expressed by mean, standard deviation, quartiles and interquartile range. Shapiro-Wilk tests served to test the normality of the continuous variables. The eight variables of interest (mTICI, mRS at 3 months, periprocedural complication rate, scopy duration, procedure duration, DAP, kerma and contrast load) were compared and dichotomized according to angiosuite type (SP or BP) using the Mann-Whitney or Fisher test for continuous or categorical variables, respectively. Then, to eliminate the effect of potential confounders, eight different models were tested. For continuous variables, a linear regression with logarithmically transformed variables was finally used after having checked that residual plots did not deviate from normality. For binary variables, we used a logistic regression. Potential confounders were determined based on a previous literature review. To retain only the most significant, a univariate analysis with the Mann-Whitney tests for quantitative variables and a Fisher exact test for qualitative variables was performed. Only variables showing a statistically significant difference were finally selected as potential confounders in the multivariate models. Multiple regression was used to adjust for age, number of pre-interventional angiograms, anesthesia type (conscious sedation or general anesthesia), MT technique (stent-retriever, contact aspiration, combination thereof), center, etiology and date of the intervention. Bonferroni correction was used to minimize inflation of type I error for the eight independent models used. We used the R Statistical Software (version 3.4.2, Foundation for Statistical Computing, Vienna, Austria) for all statistical analyses.

### Statistical analysis

Kevin ZUBER (MSc) conducted all the statistical analyses.

## Supplementary information

Supplementary information

## Data Availability

The data that support the findings of this study are available from the corresponding author upon reasonable request.
