# Supplementary material for: Safety and Effectiveness of Neuro-thrombectomy on Single compared to Biplane Angiography Systems
Source: Sci Rep. 2020 Mar 11;10:4470. doi: 10.1038/s41598-020-60851-4 (PMC7066129; doi:10.1038/s41598-020-60851-4)
Supplement: Supplementary file 1 — Supplementary information [file 41598_2020_60851_MOESM1_ESM.pdf]

Safety and Effectiveness of Neuro-thrombectomy on Single compared to Biplane Angiography Systems

Authors: Adrien Guenego, MD* [(1)]; Pascal J Mosimann, MD* [(2)]; Max Wintermark, MD, PhD [(3)]; Jeremy J Heit, MD, PhD [(3)]; Kevin Zuber, MSc [(4)]; Tomas Dobrocky, MD [(2)]; Jean Albert Lotterie, MD [(5)]; Patrick Nicholson, MD [(6)]; David G. Marcellus, BS [(3)]; Jean Marc Olivot, MD, PhD [(7)]; Nestor Gonzalez N, MD [(8)]; Raphaël Blanc, MD, MSc[(9)]; Vitor Mendes Pereira, MD, MSc [(6)]; Jan Gralla, MD, PhD [(2)]; Johannes Kaesmacher, MD[(2)], Robert Fahed, MD, MSc[(9)]; Michel Piotin, MD, PhD [(9)]; Christophe Cognard, MD [(1)]; on behalf of the **RADON** Investigators**

* Both authors contributed equally to the manuscript.

** A list of all RAdiation DOses in Neuro-thrombectomy (**RADON**) study participants is given.

[(1)] Interventional and Diagnostic Neuroradiology Department, Toulouse University Hospital, France

[(2)] Interventional and diagnostic Neuroradiology, Bern, Switzerland, Co-first author

[(3)] Interventional and Diagnostic Neuroradiology, Stanford Medical Center, California, USA

[(4)] Statistics department, Fondation Ophtalmologique Adolphe de Rothschild, Paris, France

[(5)] Stereotaxic Neurosurgery Department, Toulouse University Hospital, France

[(6)] Interventional and Diagnostic Neuroradiology, Toronto Hospital, Toronto, Canada

[(7)] Vascular Neurology, Stroke Department, Toulouse University Hospital, France

[(8)] Neurosurgery and Interventional Neuroradiology Department, Cedars Sinai Hospital, USA

[(9)] Interventional Neuroradiology, Fondation Ophtalmologique Adolphe de Rothschild, Paris, France

Statistical Analysis:

Kevin ZUBER (MSc) conducted all the statistical analyses.

Corresponding author:

Adrien GUENEGO, MD

Department of Interventional Neuroradiology,

Toulouse University Hospital

Hôpital Pierre-Paul Riquet, Place du Dr Baylac, TSA 40 031

31059 Toulouse cedex 9

FRANCE

+33 6 77 63 21 66 (Phone)

+33 5 61 77 57 42 (Fax)

adrienguenego@gmail.com (mail)

Address for correspondence :

Dr Adrien GUENEGO

Department of Interventional Neuroradiology – Pr COGNARD,

CHU Toulouse

Hôpital Pierre-Paul Riquet, Place du Dr Baylac, TSA 40 031

31059 Toulouse cedex 9

FRANCE

Manuscript type: Original Research

Words count for the structured abstract: 195

Words count for the text: 2011

References: 42

Tables: 2 (2 **Supplement Tables)**

Figures: 0

Supplementary Tables

Centers	AR name	SP/BP Dates	Maintenance	Detectors (cm) Frontal/Lateral	Field size	Frame/s	Filtration
**A**	Philips Allura Singleplane	SP 2014/2017	3/year	40/30	27	2F/sec	variable of pt. size
**A**	Philips Allura Biplane	BP 2014/2015	3/year	40/30	27/25	2F/sec	variable of pt. size
**B**	Philips Allura Singleplane	SP 2014/2016	2/year	48	27	2F/sec	variable of pt. size
**B**	Philips Allura Biplane	BP 2014/2016	2/year	48/42	27/25	2F/sec	variable of pt. size
**C**	Siemens Artis Z Biplane	BP 2014/2017	4/year	40/32	32/32	4F/sec	variable of pt. size
**D**	Siemens Artis Z Singleplane	SP 2014/2016	2/year	40x30	22	3F/sec	variable of pt. size

Supplementary Table 1. Description of angiorooms

Centers	Staff INR	Fellow	Involvement (Attending/Fellows)	Radial access	Ethics IRB
**A**	6	2	1/1	0%	Yes
**B**	5	3	1/1	1%	Yes
**C**	4	2	1/1	0%	Yes
**D**	5	3	1/0.3	0.1%	Yes

Supplementary table 2. Centers description

Supplementary Data

1. Detailed methods, angiosuites and patients characteristics

AISLVO was defined as occlusion of first portion of the middle cerebral artery (M1-MCA), second portion of the MCA (M2-MCA), internal carotid artery termination (ICA T), tandem (cervical ICA plus ICA T or M1 or M2) or Basilar Artery.

The following data were reviewed: Clinical data [center, patient sex, age (years), stroke date, weight (kg), previous modified Rankin Scale (mRS), onset National Institute of Health Stroke Scale (NIHSS), realization of a supra-aortic CT angiogram or Magnetic Resonance Imaging (MRI) angiogram previously, side of the occlusion (left, right, bilateral or midline) and level of the arterial occlusion, administration of intravenous Thrombolysis]; Thrombectomy data [type of anesthesia (general anesthesia versus conscious sedation), realization of a diagnostic angiogram to evaluate collaterals before to the thrombectomy, symptoms onset/groin delay (min), groin/recanalisation delay (min), device for thrombectomy (either with stentrievers, aspiration or combined (Solumbra) techniques), total number of passes, stenting and/or angioplasty of an extracranial vessel (carotid artery in case of Tandem lesion), successful recanalisation rate (Thrombolysis In Cerebral Infarction score (TICI) 2b/3), procedure-related complication rate (perforation, embol in new territory, hematoma on immediate post-thrombectomy to 24-hour MRI or CT), 24h NIHSS, 3 months outcome (good clinical outcome was defined as a modified Rankin score from 0 to 2), stroke etiology]; angiosuite data [angiosuite brand and type (Philips versus Siemens, mono versus biplane), Dose Area Product ($Gy.cm^2$, addition of the frontal and lateral plane doses together for the biplane), contrast load (mL, automatically extracted from the reports) and type of contrast, duration of fluoroscopy (min), Kerma (Gy)]. All patients presented an AISLVO confirmed by MRI/MRI angiogram and/or CT/CT angiogram (with at least images of the Willis Circle).

2. Dose metrics

DAP is the integral of air kerma (the energy extracted from an x-ray beam per unit mass of air in a small irradiated air volume; for diagnostic x-rays, the dose delivered to that volume of air) across the entire x-ray beam emitted from the x-ray tube. It is a surrogate measure of the amount of energy delivered to the patient[42]. Air kerma (also known as reference dose, cumulative dose, or cumulative dose at a reference point) is the air kerma accumulated at a specific point in space (the patient entrance reference point) relative to the gantry of the fluoroscopy system[42]. For C-arm fluoroscopy systems, the patient entrance reference point is a point along the central ray of the x-ray beam, 15 cm back from the isocenter toward the focal spot[43].

STROBE Statement—checklist of items that should be included in reports of observational studies

	Item No.	Recommendation	
**Title and abstract**	1	(*a*) Indicate the study's design with a commonly used term in the title or the abstract	1
		(*b*) Provide in the abstract an informative and balanced summary of what was done and what was found	4
		**Introduction**	
Background/rationale	2	Explain the scientific background and rationale for the investigation being reported	6
Objectives	3	State specific objectives, including any prespecified hypotheses	6
		**Methods**	
Study design	4	Present key elements of study design early in the paper	13
Setting	5	Describe the setting, locations, and relevant dates, including periods of recruitment, exposure, follow-up, and data collection	13
Participants	6	(*a*) *Cohort study*—Give the eligibility criteria, and the sources and methods of selection of participants. Describe methods of follow-up<br>*Case-control study*—Give the eligibility criteria, and the sources and methods of case ascertainment and control selection. Give the rationale for the choice of cases and controls<br>*Cross-sectional study*—Give the eligibility criteria, and the sources and methods of selection of participants	13
		(*b*) *Cohort study*—For matched studies, give matching criteria and number of exposed and unexposed<br>*Case-control study*—For matched studies, give matching criteria and the number of controls per case	13-14
Variables	7	Clearly define all outcomes, exposures, predictors, potential confounders, and effect modifiers. Give diagnostic criteria, if applicable	14
Data sources/ measurement	8*	For each variable of interest, give sources of data and details of methods of assessment (measurement). Describe comparability of assessment methods if there is more than one group	14-15
Bias	9	Describe any efforts to address potential sources of bias	13
Study size	10	Explain how the study size was arrived at	7
Quantitative variables	11	Explain how quantitative variables were handled in the analyses. If applicable, describe which groupings were chosen and why	14-15
Statistical methods	12	(*a*) Describe all statistical methods, including those used to control for confounding	14-15
		(*b*) Describe any methods used to examine subgroups and interactions	14-15
		(*c*) Explain how missing data were addressed	14-15
		(*d*) *Cohort study*—If applicable, explain how loss to follow-up was addressed<br>*Case-control study*—If applicable, explain how matching of cases and controls was addressed<br>*Cross-sectional study*—If applicable, describe analytical methods taking account of sampling strategy	NA
		(*e*) Describe any sensitivity analyses	NA
		**Results**	
Participants	13*	(a) Report numbers of individuals at each stage of study—eg numbers potentially eligible, examined for eligibility, confirmed eligible, included in the study, completing follow-up, and analysed	7
		(b) Give reasons for non-participation at each stage	NA
		(c) Consider use of a flow diagram	NA
Descriptive data	14*	(a) Give characteristics of study participants (eg demographic, clinical, social) and information on exposures and potential confounders	7
		(b) Indicate number of participants with missing data for each variable of interest	7
		(c) *Cohort study*—Summarise follow-up time (eg, average and total amount)	NA

Outcome data	15*	*Cohort study*—Report numbers of outcome events or summary measures over time	7
		Case-control study—Report numbers in each exposure category, or summary measures of exposure	NA
		Cross-sectional study—Report numbers of outcome events or summary measures	NA
Main results	16	(*a*) Give unadjusted estimates and, if applicable, confounder-adjusted estimates and their precision (eg, 95% confidence interval). Make clear which confounders were adjusted for and why they were included	7-8
		(*b*) Report category boundaries when continuous variables were categorized	NA
		(*c*) If relevant, consider translating estimates of relative risk into absolute risk for a meaningful time period	NA
Other analyses	17	Report other analyses done—eg analyses of subgroups and interactions, and sensitivity analyses	8
**Discussion**			
Key results	18	Summarise key results with reference to study objectives	10
Limitations	19	Discuss limitations of the study, taking into account sources of potential bias or imprecision. Discuss both direction and magnitude of any potential bias	11
Interpretation	20	Give a cautious overall interpretation of results considering objectives, limitations, multiplicity of analyses, results from similar studies, and other relevant evidence	9-10
Generalisability	21	Discuss the generalisability (external validity) of the study results	10
**Other information**			
Funding	22	Give the source of funding and the role of the funders for the present study and, if applicable, for the original study on which the present article is based	NA
